# Surgery on the Road to the Land of Promise–Ιmpact of the Refugee Crisis on the Greek Healthcare System: Results from a Surgical Department of a Tertiary Hospital

**DOI:** 10.3390/healthcare13090975

**Published:** 2025-04-23

**Authors:** Christos Damaskos, Nikolaos Garmpis, Dimitrios Lamprinos, Gregory Kouraklis, Dionysios Prevezanos, Anna Garmpi, Miltiadis-Panagiotis Papandroudis, Iason Psilopatis, Dimitrios Papoutsas, Georgios Marinos, Stavros Kourlakis, Eleni I. Effraimidou

**Affiliations:** 1 Department of Emergency Surgery, Laiko General Hospital, 11527 Athens, Greece; 2 N.S. Christeas Laboratory of Experimental Surgery and Surgical Research, Medical School, National and Kapodistrian University of Athens, 11527 Athens, Greece; nikosg22@hotmail.com; 3 Department of Surgery, Sotiria General Hospital, 11527 Athens, Greece; papdr@yahoo.com; 4 Emergency Care Department, Laiko General Hospital, 11527 Athens, Greece; dimitrislamprinos@gmail.com; 5 Medical School, National and Kapodistrian University of Athens, 11527 Athens, Greece; gkouraklis@hotmail.com; 6 Second Department of Propedeutic Surgery, Laiko General Hospital, Medical School, National and Kapodistrian University of Athens, 11527 Athens, Greece; prevedio@hotmail.com (D.P.); sta.kourlakis@gmail.com (S.K.); 7 8th Department of Internal Medicine, Ygeia General Hospital, 15123 Athens, Greece; annagar@windowslive.com; 8 Medical School, University of Plovdiv, 4002 Plovdiv, Bulgaria; manchmilt@gmail.com; 9 Department of Obstetrics and Gynecology, University Erlangen Hospital, 91012 Erlangen, Germany; iason.psilopatis@usb.ch; 10 Department of Hygiene, Epidemiology and Medical Statistics, Medical School, National and Kapodistrian University of Athens, 11527 Athens, Greece; gmarino@med.uoa.gr; 11 First Surgical Department, University Hospital of Alexandroupolis, Democritus University of Thrace, Dragana, 68100 Alexandroupolis, Greece; eeffraem@med.duth.gr

**Keywords:** surgery, refugee crisis, financial implication, national healthcare system, NHS, Greece

## Abstract

Background/Objectives: The surge in migration from the Middle East and North Africa due to conflicts has significantly impacted healthcare systems, particularly in Greece. This study investigates how the sharp increase in refugees and migrants after July 2015 has strained the surgical departments of the Greek National Health System (NHS). Methods: A retrospective analysis was conducted on 229 patients treated at the emergency department of a public hospital in Athens, Greece. Data were compared between two periods: January 2012–July 2015 (pre-July 2015) and July 2015–December 2018 (post-July 2015), with July 2015 chosen as the cutoff due to a significant influx of immigrants during that time. Results: Patients’ demographic details, diagnoses, and surgical interventions were analyzed. Results indicated a significant rise in surgical cases, with 72.5% of patients requiring procedures, notably for appendicitis (23.6%), cholecystitis (10.9%), lower extremity thrombophlebitis (9.6%), perianal abscess (8.3%), and inguinal hernia (5.7%). Post-July 2015, there was a notable increase in perianal abscess (12.2%), inguinal hernia (8.4%), and cholelithiasis (6.1%). However, the average hospital stay of 3.9 days remained unchanged. Conclusions: The findings reveal the profound economic and operational pressures on the NHS during the refugee crisis, highlighting the urgent need for resource optimization and policy reforms. Future studies should address long-term healthcare impacts to support more sustainable healthcare models amidst ongoing and future migration challenges.

## 1. Introduction

The global migration crisis has had profound repercussions on healthcare systems worldwide, particularly within the emergency departments of public hospitals. The rising instability and insecurity in some parts of the Middle East and North Africa due to military conflicts has consequently led to an increase in the number of people trying to reach the European Union (EU). The most used escape routes are the Mediterranean Passage–Southeastern Mediterranean route through Turkey to Greece and the central Mediterranean route through Egypt and Libya to Italy [1]. According to the United Nations High Commissioner for Refugees (UNHCR), over 850,000 arrivals of refugees have been recorded to Greece, with more than half after the 1st of July 2015. From January until August of this year, there was an increase in arrivals in Greece by 850% compared to the same period in 2014 [2]. The immigrants came mainly from Syria, Afghanistan, and Iraq [3]. Obviously, these increased numbers of people entering the country–with some potential patients among them–caused a lot more strain on the national health system (NHS). The main health problems of these immigrants can be categorized inro trauma, respiratory tract infections, skin diseases, mental and social problems, vaccine preventable diseases, gastrointestinal diseases, and non-communicable diseases (NCDs) [4,5,6].

In addition to crises in the Middle East and North Africa, ongoing conflicts such as the Russia–Ukraine war have further exacerbated global displacement. Millions of Ukrainians have been forced to flee to neighboring countries, significantly increasing the strain on their healthcare systems. This surge of refugees has resulted in an uptick in hospital admissions, particularly for trauma care, chronic disease management, and mental health services. The NHS of host countries face immense pressure to accommodate both local populations and displaced individuals, often with limited resources. As seen in Greece, sudden increases in refugee arrivals overwhelm emergency departments, highlighting the global challenge of providing equitable and sustainable healthcare in the face of persistent geopolitical conflicts.

The Greek NHS provides healthcare services through a network of public/state providers and contracted private providers of primary, hospital, and ambulatory care with the aim to ensure disease prevention and the promotion, preservation, improvement, recovery, and protection of health. The presence of private providers is more obvious in primary care, especially in diagnostic technologies, private physicians’ practices, and pharmaceuticals. As far as emergencies are concerned, one can seek treatment directly in a public hospital. Urgent medical care is always free of charge. Medical services through appointment with a doctor in a public hospital are also free of charge. In pharmacies, there is usually a co-payment of 25% of the cost of medicinal products if there is a prescription from a doctor. Some patient groups, such as the chronically ill, pregnant women, and the uninsured, receive medicines free of charge or pay a reduced co-payment [7]. If there is a need for hospitalization or in-hospital treatment, the cost of each case is calculated through the diagnosis-related groups (DRGs), a system which combines the diagnosis (International Classification of Disease, ICD-10), the duration of hospitalization, the medical procedure, and counts the total expenses [8]. It should be mentioned that the Greek NHS faced significant pressure due to the government-debt crisis of the last decade [9]. Greek hospitals faced significant challenges in meeting the healthcare demands of both local residents and migrants, largely due to limited medical and human resources amid the economic crisis [10,11,12]. Additionally, language barriers and cultural differences, particularly gender-related in healthcare interactions, further hindered access to medical services [13].

The purpose of paper is to investigate how the influx of refugees and migrants to Greece has affected the surgical department of the NHS after the peak of July 2015.

## 2. Materials and Methods

This study involved a retrospective review of medical records from 229 patients who attended the emergency department of the surgical unit in a public hospital during two distinct time periods. The patients were classified as refugees or migrants based on their documented nationality and legal status. The hospital, situated in a region with a significant influx of refugees and migrants, was selected due to the high volume of such patients it serves. The purpose of the study was to assess the effects of the increased migration flow on the surgical services of the Greek NHS following July 2015. The study was conducted in accordance with ethical guidelines and the guidelines of the Declaration of Helsinki and approval was obtained from the Ethics Committee of Laiko General Hospital (protocol code: 90/18/03/2025). Additionally, written permission was secured prior to accessing the data.

The study period was divided into two equal intervals for comparison: the first from January 2012 to June 2015 and the second from July 2015 to December 2018. Data extracted from medical records included demographic details such as age and gender. Gender was categorized as male or female, while age was recorded as a continuous variable and reported as mean ± standard deviation (SD). The duration of hospital stays, measured in days, was also recorded and used as a measure of resource utilization and hospital workload.

The need for surgical procedures was recorded and analyzed to identify trends or differences between the two time periods. Furthermore, the patients’ diagnoses were grouped into specific categories, including appendicitis, cholecystitis, lower extremity thrombophlebitis, perianal abscess, and inguinal hernia, among others. This categorization facilitated an evaluation of shifts in disease patterns and surgical demands associated with the influx of refugees and migrants.

A range of descriptive and inferential statistical methods was employed to analyze the data. Continuous variables, such as age and hospital stay duration, were summarized using mean and SD, while categorical data, including gender distribution, diagnosis type, and surgical intervention rates, were reported as absolute frequencies and percentages. Statistical analysis was performed by the *t*-test which was used to compare continuous variables, such as age and length of hospitalization, between the two periods; the Chi-square test (χ^2^) which assessed the relationships between categorical variables, including gender, the need for surgery, and diagnosis type, in relation to the two time periods; and the z-test that compared the prevalence rates of specific diseases between the pre- and post-July 2015 groups.

The threshold for statistical significance was set at *p* = 0.05, and a Bonferroni correction was applied to address the issue of multiple comparisons, ensuring robust statistical validity. All analyses were conducted using IBM SPSS Statistics 27 software. This methodology provided a detailed assessment of the impact of increased migration on the surgical workload and resources of the public hospital.

## 3. Results

This study included a total of 229 patients, of whom 98 (42.8%) received treatment before July 2015. During this earlier period, the emergency department recorded an incidence of 228.2 cases per 100,000 patients out of a total of 42,952 visits. The remaining 131 patients (57.2%) were treated after July 2015, corresponding to an increased rate of 366.3 cases per 100,000 patients among 35,765 emergency department visits (Table 1).

The participants had an average age of 41.4 years, with a SD of ±15.9 years (Table 2).

The group comprised 133 males (58.1%) and 96 females (41.9%) (Table 3). Analysis showed no statistically significant differences in either gender distribution or mean age between the two periods (χ^2^ test *p* = 0.289; *t*-test *p* = 0.327).

**Figure 1:**
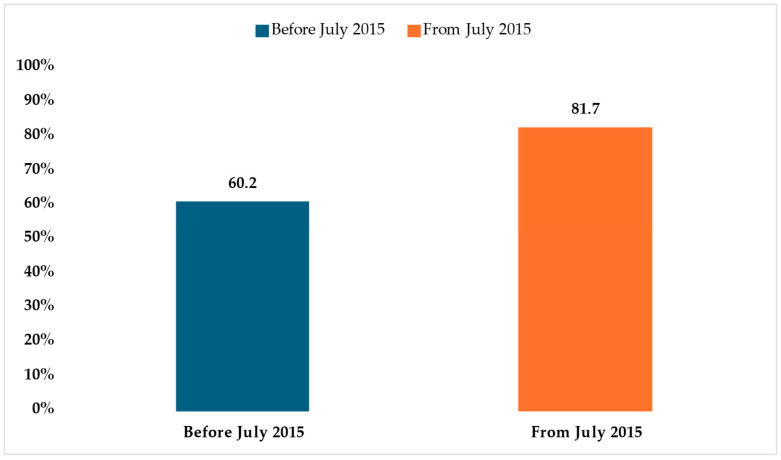
Number of patients who underwent surgery by period.

Surgical interventions were performed in 72.5% of the cases (n = 220), with a marked increase in the post-July 2015 group (81.7%, n = 107) compared to the pre-July 2015 group (60.2%, n = 59) (Table 4). This difference was highly significant (χ^2^ test *p* < 0.001) (Figure 1). These findings reflect a notable shift in clinical management patterns over time.

**Figure 2:**
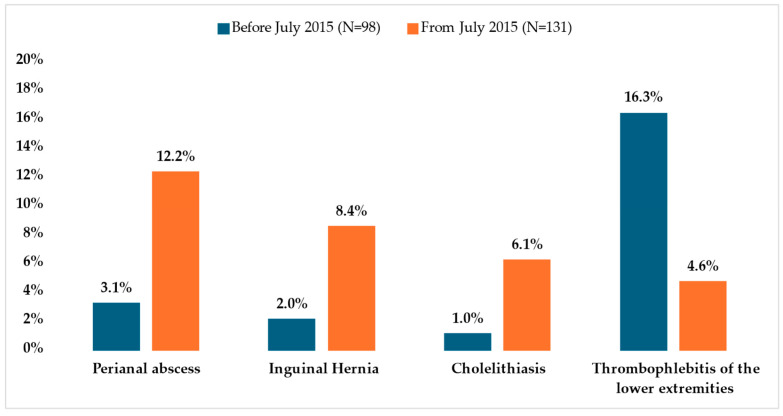
Patients with the most frequently observed diagnoses who underwent surgery by period.

The most frequently observed diagnoses across all patients were appendicitis (n = 54, 23.6%), cholecystitis (n = 25, 10.9%), lower extremity thrombophlebitis (n = 22, 9.6%), perianal abscess (n = 19, 8.3%), and inguinal hernia (n = 13, 5.7%) (Table 5 and Table 6). A statistically significant association was identified between the prevalence of these conditions and the time period in which treatment was administered (χ^2^ test *p* = 0.036). Specifically, post-July 2015, patients exhibited higher rates of perianal abscess (12.2%), inguinal hernia (8.4%), and cholelithiasis (6.1%) compared to those treated earlier (3.1%, 2.0%, and 1.0%, respectively). These differences were statistically significant, with z-test *p*-values of 0.012, 0.039, and 0.05, respectively. Conversely, lower extremity thrombophlebitis was more prevalent before July 2015 (16.3%) than after (4.6%), with a z-test *p*-value of 0.003 (Figure 2). For other conditions, no statistically significant variations were observed between the two time frames.

The mean hospital stay for the entire cohort was 3.9 days (±4.0 days). This duration did not significantly differ between the two periods (*t*-test *p* = 0.212) (Table 7). Overall, the study highlights significant temporal trends in disease presentation and management strategies, underscoring changes in clinical practices and patient characteristics over time.

## 4. Discussion

The unprecedented influx of refugees and migrants into Greece, particularly following July 2015, placed a considerable burden on the country’s NHS. This surge was largely due to European border closures, which left a significant number of migrants and asylum seekers stranded in Greece. Consequently, the NHS faced immense challenges in addressing the healthcare needs of both the local population and the newly arrived migrants. These difficulties were exacerbated by the limited resources available to the NHS, making it increasingly challenging to respond effectively to the heightened demand [12,14].

As indicated in this study, there was a statistically significant rise in specific health conditions among the refugee population, such as perianal abscesses, inguinal hernias, cholelithiasis, and thrombophlebitis. These findings align with expectations given the harsh living conditions migrants often endure during their journeys. Prolonged exposure to poor sanitation, overcrowding, and limited access to basic hygiene can lead to the development of infections, including abscesses. Similarly, the physical demands of migration, such as carrying heavy loads and extended periods of walking, can exacerbate or precipitate conditions like inguinal hernias. Additionally, underlying health vulnerabilities, combined with limited physical activity or prolonged immobility during the journey, may contribute to the development of thrombosis in the lower extremities. However, the study observed a reduction in thrombophlebitis rates after July 2015, which can be explained by the shift in migration routes. Post-July 2015, the majority of refugees arrived in Greece by boat via sea routes, which involved less physical exertion compared to the land routes predominant before this period. Furthermore, the poor nutritional intake of migrants, both in quality and quantity, likely contributed to the increased incidence of cholecystitis.

In terms of treatment, most of these conditions were managed surgically, except for thrombophlebitis, which was treated conservatively using medications such as anticoagulants. The increase in surgeries required for conditions like abscess drainage, hernia repair, and cholecystectomy significantly contributed to the strain on the healthcare system. While conservative management of thrombophlebitis might suggest an extended length of hospitalization, the study found no statistically significant difference in average hospital stays between the two periods.

The economic impact of this increased surgical workload on the NHS is evident, particularly given the limited resources and pre-existing financial challenges faced by the Greek healthcare system. However, accurately quantifying the financial burden is complex due to the limitations of the NHS’s costing system. In Greece, healthcare costs are determined based on DRGs, which apply standard costs to medical procedures without accounting for individual variations. Factors such as hospitalization duration, medication usage, diagnostic tests, and consumable resources are not considered, resulting in a uniform costing model that does not reflect the actual financial implications of each case. Additionally, this standardized approach may lead to inaccuracies in evaluating the quality of care provided [15].

The economic crisis in Greece further compounded these challenges, leading to reduced healthcare expenditure and a weakened NHS that struggled to meet the increased demands associated with the refugee crisis [16]. Although healthcare providers were committed to delivering care, they faced significant obstacles, including insufficient resources, inadequate training, and a lack of cultural competence to address the unique needs of a multicultural patient population [11,17,18,19].

When comparing Greece’s experience to other healthcare systems managing refugee populations, parallels and differences become apparent. For instance, the Nyarugusu Refugee Camp in Tanzania primarily provided surgical care focusing on acute conditions like hernia repairs, exploratory laparotomies, appendectomies, hemorrhoidectomies and lipoma excisions, reflecting the constraints of resource-limited settings [20,21]. Similarly, Turkey, which managed a significant influx of Syrian refugees, faced disparities in access to surgical services. For example, cesarean section rates were lower among Syrian refugee women compared to Turkish nationals, influenced by cultural differences and limited healthcare access [22]. Furthermore, injuries and burns account for the vast majority of surgical pathologies in refugees [23,24]. In contrast, Greece faced a unique challenge of addressing the increased demand for adult surgical interventions, such as appendectomies and cholecystectomies, during an economic crisis that further strained its healthcare system.

Global studies underscore the increasing burden of surgical needs in humanitarian settings. These findings highlight the necessity of capacity building in healthcare systems, including investments in surgical infrastructure and training to manage both acute and chronic conditions effectively [25]. The challenges faced by Greece mirror those observed in other contexts, emphasizing the urgent need for strengthened healthcare infrastructure, enhanced training for surgical teams, and comprehensive policies to address the complex healthcare needs of refugee populations.

Except for surgical problems, other medical problems have been raised since 2015. Refugees face a significant burden of health issues due to the protracted asylum process and poor living conditions. In one study, the prevalence of major depressive disorder was reported as 44%, with risk factors including prolonged asylum application periods, being female, and having more children, while marriage was a protective factor [26]. Chronic illnesses such as hypertension, diabetes, and other NCDs were also common, although systematic screening efforts were limited due to resource constraints and frequent population movements [27]. Acute respiratory and gastrointestinal infections were particularly prevalent, exacerbated by overcrowded living conditions, insufficient sanitation, and environmental exposures [26,27]. Skin and soft tissue conditions, including scabies and other parasitic infestations, were frequently reported, with reinfections being common due to disrupted hygiene facilities in camps [27].

Humanitarian organizations were critical in providing primary healthcare services and referrals for specialist care, filling essential gaps left by the strained Greek healthcare system. Dental care emerged as a significant unmet need, representing 25% of all referrals, despite comprising only 5% of consultations, underscoring the lack of focus on dental health in humanitarian guidelines [27]. Furthermore, mental health and psychosocial support services were underfunded and culturally misaligned, limiting their effectiveness. Refugees’ psychosocial issues were compounded by prolonged asylum processes and inadequate mental health services, reflecting systemic barriers to access. The Greek NHS, already burdened by the country’s economic crisis, faced immense challenges in integrating refugee healthcare. Although refugees were entitled to free healthcare under Greek law, the need for social security numbers created barriers to accessing non-emergency services [27]. Humanitarian organizations helped to reduce the impact on the national system by embedding specialists in clinics and streamlining referrals. However, language barriers, logistical challenges, and inadequate funding for long-term health integration continued to hinder access. The findings emphasize the importance of coordinated efforts between humanitarian actors and the NHS to provide equitable, sustainable healthcare for both refugees and the host population, with a focus on improving coordination, resource allocation, and culturally sensitive care [26,27].

In addition, the implementation of the EU–Turkey agreement in March 2016 marked a turning point in the experiences of Syrian refugees in Greece, significantly reducing migration flows but leaving approximately 60,000 individuals stranded in camps under precarious conditions while awaiting asylum processing. This prolonged encampment shifted health needs from acute issues, such as hypothermia and injuries, to chronic conditions, mental health disorders, and heightened risks of gender-based violence (GBV). Stakeholders reported a deterioration in mental health, with refugees frequently exhibiting symptoms of depression, anxiety, and post-traumatic stress disorder, largely due to prolonged uncertainty and poor living conditions. Women and minors faced increased risks of GBV in overcrowded and insecure camps, where inadequate protection measures compounded vulnerabilities. Although healthcare provision evolved to address chronic and mental health needs, systemic gaps, including insufficient referral mechanisms and limited access to specialized care, persisted [13].

Another publication highlights the immense strain that the influx of Syrian refugees has placed on Jordan’s NHS, particularly in the management of chronic health conditions. Among Syrian refugees diagnosed with NCD, such as hypertension, diabetes, and cardiovascular disorders, over 84% sought medical care, with the majority relying on public healthcare facilities (53.9%), while private providers and non-governmental organizations accounted for smaller shares of service delivery. This increased demand for chronic disease management has exerted significant pressure on Jordan’s public health infrastructure, especially in urban centers like Amman, which house the largest refugee populations. Such dependence on public sector services is further exacerbated by the limited availability of healthcare resources, insufficient funding, and the economic strain experienced by both the Jordanian government and the refugee communities [28].

Moreover, financial barriers remain a critical impediment to healthcare access for Syrian refugees in Jordan. While refugees initially benefited from free access to public healthcare services, policy changes implemented in late 2014 introduced subsidized rates equivalent to those paid by uninsured Jordanians. Out-of-pocket costs for consultations and essential medications have further restricted the ability of refugees to maintain continuity of care, particularly for conditions requiring long-term management. These challenges underscore the urgent need for targeted investments in Jordan’s NHS, coupled with sustained international support, to mitigate the healthcare burden and ensure equitable access to services for both refugee and host populations [28].

The migration of refugees to the United States (U.S.), like Greece, places significant demands on NHS, though the challenges differ due to variations in economic capacity, healthcare infrastructure, and policy frameworks. The U.S. healthcare system employs proactive health interventions, such as pre-departure medical screenings and vaccinations, to mitigate the immediate burden on domestic healthcare providers. For example, pre-departure programs for U.S.-bound refugees effectively reduced helminthic infections from 67% to 12% and halved the prevalence of moderate-to-severe anemia through targeted treatments and nutritional support [29]. In contrast, Greece lacks comparable pre-migration interventions, leaving its NHS to handle acute and chronic conditions upon arrival. This disparity underscores the importance of upstream health measures, as demonstrated by the U.S. model, which alleviates healthcare burdens post-arrival [29].

However, both countries face systemic challenges in managing the long-term healthcare needs of refugees. In the U.S., variability in state-level domestic medical examinations leads to unequal access to follow-up care for conditions like hepatitis B and anemia, similar to Greece’s fragmented approach to healthcare delivery for refugees [29]. Additionally, both systems struggle with the effective integration of mental health services, a critical need for displaced populations dealing with trauma and chronic stress. While the U.S. benefits from its economic resources to implement structured interventions, Greece’s financial and logistical constraints exacerbate the strain on its already overstretched NHS. International collaboration and resource allocation are crucial for both countries to ensure equitable healthcare access for refugee populations and to address disparities in their healthcare systems.

To summarize, the health challenges posed by refugee crises worldwide emphasize the need for targeted, context-specific interventions to strengthen each country’s NHS. As seen in Greece, Jordan, and the U.S., the integration of refugee health services into national health frameworks presents unique challenges related to resource allocation, access to care, and the management of acute and chronic conditions. While Greece and Jordan primarily struggle with financial and logistical constraints, the U.S. model of pre-departure health interventions demonstrates the importance of proactive measures to mitigate healthcare burdens. However, all three systems highlight the critical need for enhanced mental health services, improved cultural competence, and international collaboration to address the complex needs of refugee populations effectively. By prioritizing investments in healthcare infrastructure, fostering public–private partnerships, and leveraging global support, countries can develop sustainable solutions that not only cater to refugees but also strengthen healthcare delivery for host communities.

In this direction, and taking into account future waves of mass migration given the current geopolitical conditions such as the wars in the Ukraine and the Middle East, the creation of health data registries is considered particularly important and beneficial for healthcare systems [30].

### Limitations of the Study

Despite the clear evidence that the influx of refugees after July 2015 placed a significant financial burden on public health services, the true cost remains difficult to quantify due to systemic limitations in cost reporting. Moreover, the retrospective nature of this study introduces potential selection bias, and its focus on a single public hospital may limit the generalizability of the findings.

Also, our study includes only emergencies and no other entities such as infections (helminthiasis, amoebiasis, salmonellosis, etc.) that can lead to surgical therapy if left untread [31].

Additionally, the study does not account for the long-term healthcare outcomes of patients or the broader healthcare demands, such as mental health services, preventative care, and the management of chronic diseases, which are crucial components of the overall healthcare burden. Furthermore, the lack of comparative data from the local population during the same period limits the ability to isolate the specific impact of the refugee influx on the Greek NHS. Last but not least, we have to mention that our study includes only data from a tertiary hospital in the capital. It is likely that the results may differ in health facilities near the country’s borders or refugee reception centers.

Future research should aim to evaluate non-surgical healthcare demands, incorporate long-term patient outcomes, and involve multiple healthcare facilities to provide a more comprehensive assessment of the refugee crisis’s impact on the Greek NHS.

## 5. Conclusions

This study highlights the multifaceted impact of refugee crises on healthcare systems, particularly in the context of urgent surgical care demands, resource allocation, and overall system efficiency. Surgical departments face systemic challenges such as inadequate infrastructure, funding shortages, and the need for specialized training to accommodate diverse patient needs. While the study does not aim to provide a precise numerical estimate of the economic burden, it offers valuable insights into the effects of migration on healthcare systems. By documenting the rising patient load in Greek hospitals, it contributes to a better understanding of how large-scale migration strains national health services both financially and structurally.

Additionally, this study underscores the need for further research on healthcare demands beyond surgical clinics, including non-surgical departments, to provide a more comprehensive perspective on the long-term impact of migration on healthcare systems. In the wake of the COVID-19 pandemic and ongoing global conflicts, the challenges faced by national healthcare systems in managing migrant health demands remain highly relevant.

### Practical Implications

The findings of this study underscore several critical interventions necessary to strengthen the resilience of healthcare systems in response to large-scale migration. Strengthening emergency care infrastructure is of paramount importance, as it enhances the capacity of healthcare facilities to manage increased patient volumes and ensures timely access to medical interventions during refugee crises. The integration of cultural competency training for healthcare professionals is essential to improving communication, fostering trust, and promoting patient-centered care tailored to the diverse linguistic and cultural backgrounds of migrant populations.

Furthermore, enhancing collaboration with international agencies is crucial for optimizing resource allocation, facilitating coordinated responses, and aligning policy frameworks to provide sustained support for healthcare systems operating under heightened demand. Addressing the socio-economic determinants of refugee health, including housing instability, employment barriers, and limited healthcare access, is vital in mitigating long-term healthcare burdens and improving health outcomes within displaced populations. Additionally, the establishment of comprehensive migrant health databases would contribute to more effective healthcare planning, equitable resource distribution, and efficient cost management by enabling data-driven decision-making and policy development.

These findings provide valuable insights for policymakers, healthcare administrators, and public health officials striving to enhance the preparedness, adaptability, and responsiveness of healthcare systems in the face of ongoing and future migration-related challenges. By implementing these strategic interventions, healthcare systems can better navigate the complexities of large-scale migration, ensuring equitable access to high-quality medical care while maintaining overall system efficiency and sustainability.

This study highlights the complex effects of refugee crises on healthcare, focusing on urgent surgical demands and impacts on resources and efficiency. Surgical departments face systemic challenges like inadequate infrastructure, funding issues, and the need for specialized training for varied patient needs. The study identifies opportunities for interventions to improve healthcare resilience. Key steps include investing in emergency care, integrating cultural competency training, and collaborating with international agencies. Additionally, addressing refugees’ socio-economic health determinants can reduce long-term healthcare burdens. 

While the study does not strive to provide a precise numerical estimate of the economic burden, it is an important study in understanding the impact of migration on healthcare systems. Its value lies in providing insights to help health systems prepare for the effects of large-scale migration, both financially and structurally. This study flags the need for research on healthcare demands beyond surgical clinics. It lays a foundation for further research on attendance and care in non-surgical departments, helping move toward a more complete understanding of the migration crisis since 2015. This kind of study is very important to inform policy and strengthen health systems facing similar global challenges. The subject of the current study is of high relevance in a post-pandemic situation and in ongoing wars. Many countries’ NHS are facing difficulties in offering health care services. Databases for migrants need to be developed to maintain a balance of enhanced services alongside the limitations associated with their cost.

## Figures and Tables

**Table 1:** Number and percentage of patients per year.

Year		N		%	
2012		35		15.3	
2013		38		16.6	
2014		17		7.4	
2015	Before July	17	8	7.4	3.5
	From July		9		3.9
2016		39		17.0	
2017		49		21.4	
2018		34		14.8	
Total		229		100.0	

N: number of patients.

**Table 2:** Age of patients by period.

Age (Y)	Time Period		Total
	Before July 2015	From July 2015	
Mean value	42.6	40.5	41.4
Standard deviation	16.8	15.2	15.9
N	98	131	229

Y: years; N: number of patients.

**Table 3:** Gender of patients by period.

Gender	Time Period					
	Before July 2015		From July 2015		Total	
	N	%	N	%	N	%
Male	53	54.1	80	61.1	133	58.1
Female	45	45.9	51	38.9	96	41.9
Total	98	100.0	131	100.0	229	100.0

N: number of patients.

**Table 4:** Type of therapy by period.

Therapy	Time Period					
	Before July 2015		From July 2015		Total	
	N	%	N	%	N	%
Surgical	59	60.2	107	81.7	166	72.5
Conservative	39	39.8	24	18.3	63	27.5
Total	98	100.0	131	100.0	229	100.0

N: number of patients.

**Table 5:** Diseases by period.

Disease	Time Period					
	Before July 2015		From July 2015		Total	
	N	%	N	%	N	%
Appendicitis	20	20.4	34	26.0	54	23.6
Cholecystitis	15	15.3	10	7.6	25	10.9
Lower extremity thrombophlebitis	16	16.3	6	4.6	22	9.6
Perianal abscess	3	3.1	16	12.2	19	8.3
Inguinal hernia	2	2.0	11	8.4	13	5.7
Hemorrhoids	5	5.1	4	3.1	9	3.9
Cholelithiasis	1	1.0	8	6.1	9	3.9
Stomach perforation	3	3.1	5	3.8	8	3.5
Lower limb gangrene	5	5.1	2	1.5	7	3.1
Breast cancer	3	3.1	3	2.3	6	2.6
Abdominal wall injury	2	2.0	4	3.1	6	2.6
Perianal fistula	2	2.0	3	2.3	5	2.2
Intestinal obstruction syndrome	1	1.0	4	3.1	5	2.2
Abdominal abscess	2	2.0	2	1.5	4	1.7
Large intestine cancer	0	0.0	3	2.3	3	1.3
Varicose veins	2	2.0	1	0.8	3	1.3
Umbilical hernia	0	0.0	3	2.3	3	1.3
Pilonidal cyst	1	1.0	1	0.8	2	0.9
Postoperative hernia	2	2.0	0	0.0	2	0.9
Upper limb injury	1	1.0	1	0.8	2	0.9
Liver injury	1	1.0	1	0.8	2	0.9
Thoracic wall injury	1	1.0	1	0.8	2	0.9
Head injury	1	1.0	1	0.8	2	0.9
Abdominal bleeding	1	1.0	1	0.8	1	0.4
Large intestine perforation	0	0.0	0	0.0	1	0.4
Diverticulitis	0	0.0	1	0.8	1	0.4
Carotid embolism	0	0.0	1	0.8	1	0.4
Liver abscess	0	0.0	1	0.8	1	0.4
Pancreatic cancer	0	0.0	1	0.8	1	0.4
Liver cirrhosis	1	1.0	0	0.0	1	0.4
Mesentery lymphoma	1	1.0	0	0.0	1	0.4
Femoral hernia	0	0.0	1	0.8	1	0.4
Gastrointestinal foreign body	1	1.0	0	0.0	1	0.4
Ovary tumor	1	1.0	0	0.0	1	0.4
Aneurysm rupture	1	1.0	0	0.0	1	0.4
Large intestine twist	1	1.0	0	0.0	1	0.4
Duodenum injury	0	0	1	0.8	1	0.4
Pancreas injury	1	1.0	0	0.0	1	0.4
Facial injury	1	1.0	0	0.0	1	0.4
Total	98	100.0	131	100.0	229	100.0

N: number of patients.

**Table 6:** Diseases category by period.

Disease	Time Period					
	Before July 2015		From July 2015		Total	
	N	%	N	%	N	%
Trauma/bleedings	9	9.2	9	6.9	18	7.9
Viscera perforation	3	3.1	6	4.6	9	3.9
Anus/coccyx disorders	11	11.2	24	18.3	35	15.3
Hepatobiliary tract disorders	17	17.3	18	13.7	35	15.3
Cancers	5	5.1	7	5.3	12	5.2
Abdominal inflammations	22	22.4	38	29.0	60	26.2
Hernias ^1^	4	4.1	15	11.5	19	8.3
Vascular disorders ^2^	24	24.5	10	7.6	34	14.8
Other	3	3.1	4	3.1	7	3.1
Total	98	100.0	131	100.0	229	100.0

N: number of patients; ^1^ *z*-test, *p*-value = 0.046; ^2^ *z*-test, *p*-value < 0.001.

**Table 7:** Hospitalization duration by period.

Hospitalization (D)	Time Period		Total
	Before July 2015	From July 2015	
Mean value	4.3	3.6	3.9
Standard deviation	3.6	4.2	4.0
N	98	131	229

N: number of patients.

## Data Availability

Data is unavailable due to privacy or ethical restrictions.

## Abbreviations

The following abbreviations are used in this manuscript:

EU	European Union
UNHCR	United Nations High Commissioner for Refugees
NHS	National Health System
NCD	Non-Communicable Disease
DRG	Diagnosis-Related Group
ICD	International Classification of Disease
SD	Standard Deviation
GBV	Gender-Based Violence
U.S.	United States
